# Field note use in family medicine residency training: learning needs revealed or avoided?

**DOI:** 10.1186/s12909-021-02883-6

**Published:** 2021-08-27

**Authors:** Nicole Zaki, Teresa Cavett, Gayle Halas

**Affiliations:** 1 Max Rady College of Medicine, Rady Faculty of Health Sciences, University of Manitoba, Winnipeg, Manitoba Canada; 2 Department Family Medicine, University of Manitoba, Winnipeg, Manitoba Canada

**Keywords:** Medical education, Field notes, Residency, Competency-based medical education

## Abstract

**Background:**

Field notes (FNs) are used in Family Medicine residency programs to foster reflective learning and facilitate formative assessment. Residents assess their strengths and weaknesses and develop action plans for further improvement. This study explored the use of FNs in the University of Manitoba’s Family Medicine residency program 5 years after their implementation.

**Methods:**

This multi-method study examined 520 FNs from 16 recent graduates from the University of Manitoba Family Medicine residency program. Quantitative analysis (frequencies and means) enabled descriptions and comparisons between training sites. Four themes emerged from inductive content analysis highlighting common ideas reflected upon.

**Results:**

Residents displayed cyclical variation in the FN generation over 2 years. Eight of the 99 Priority Topics (addressing complex psychosocial issues) were not captured in this data set. The domains of Care of First Nations, Inuit, and Metis; Care of the Vulnerable and Underserved; and Behavioural Medicine and the CanMEDS-FM roles of FM – Procedural Skill, Leader/Manager, and Professional were less frequently reflected upon. Four themes (*Patient-Centered Care, Patient Safety, Achieving Balance,* and *Confidence)* were identified from qualitative analysis of residents’ narrative notes.

**Conclusions:**

Vygotsky’s Sociocultural Theory of Cognitive Development was proposed as a lens through which to examine factors influencing resident learning. Residents’ discomfort with certain topics may lead to avoidance in reflecting upon certain competencies in FNs, impacting skill acquisition. Further research should explore factors influencing residents’ perceptions FNs and how to best assist residents in becoming competent, confident practitioners.

## Background

Canadian Family Medicine residency programs are entrusted with ensuring the provision of competency-based education so as to prepare Family Medicine residents to take on the role of generalists. Residents must develop competence in managing a wide range of patient concerns and pathologies over a relatively short two-year residency. As such, educators are tasked with multiple challenges in ensuring well-rounded and impactful educational experiences [1, 2]. Educators must provide learners with authentic learning opportunities, frequent formative feedback, and regular summative assessments during residency [1]. During their residencies, learners will be at various stages with respect to their confidence and ability to practice independently and as such, educational experiences must vary between individuals in order to adequately prepare for future practice. It is vital to ensure that residency education is learner-centered through discussion and collaboration with residents to elicit and address residents’ personal learning needs.

Family Medicine education in Canada continues to evolve to meet residents’ and society’s needs. Ensuring the competence of residents has always been a prominent goal of residency programs as Family Medicine educators continually seek to optimize educational opportunities that will best prepare residents to be care providers across the lifespan of their patients by the end of the two-year program [2, 3]. The Competency-Based Medical Education (CBME) model aims to improve the provision of feedback and encourage continuing education by promoting learning through outcomes-based reflection by residents [4] with guidance from their supervisors. Outcomes are established based on the expected competence of residents at their level of training [4]. Residents must track and document these learning experiences, feedback, and assessments to demonstrate that they have had sufficient opportunity to achieve competence [1, 5, 6]. Furthermore, residents must develop the ability to accurately self-reflect on their clinical performance for continued quality improvement and professional development. Finally, educators must continuously assess learners’ abilities and provide constructive feedback throughout the program in order to prepare learners for future independent practice [2].

One tool used to promote reflective practice is the Field Note (FN). FNs are short reflective narratives authored by residents to encourage self-reflection and feedback by preceptors [1, 5–10]. They have been widely implemented by Family Medicine programs across Canada [6] and have recently been recommended by the College of Family Physicians of Canada (CFPC) as a best practice in CBME [1]. FNs provide residents and preceptors with an opportunity to examine a resident-patient encounter through a variety of contexts and lenses [1, 5]. Together, the resident and preceptor debrief a clinical encounter, identify the resident’s strengths and areas of weakness, and generate a plan for improvement [5, 11]. The overall intent of FNs is to generate discussion and feedback between residents and their preceptors, while additionally providing documentation of residents’ progress towards competency [1, 5]. FNs engage both residents and supervisors in appraising residents’ skills in a variety of domains, and in identifying learning gaps while formulating plans to address these deficits [5]. Accordingly, FNs facilitate the development of learner-centered competency such that residents will become skilled in evaluating their confidence and competence in both current and future practice [4].

Canadian Family Medicine residency education is comprised of 2 years of clinical and didactic training. At the University of Manitoba, the Family Medicine residency program is divided into nine streams which fall into three general groups: Urban, Rural, and Northern Remote [12–14]. Within the Family Medicine streams, faculty preceptors work directly with the residents in an apprenticeship model, which affords the opportunity to provide comprehensive feedback to the resident on their performance and capabilities. Physician faculty observe residents’ progress and provide ongoing feedback throughout the program with input from interprofessional faculty members (e.g. clinical nurse specialists, pharmacists, social workers) affiliated with the Family Medicine clinical teaching units.

**Fig. 1:**
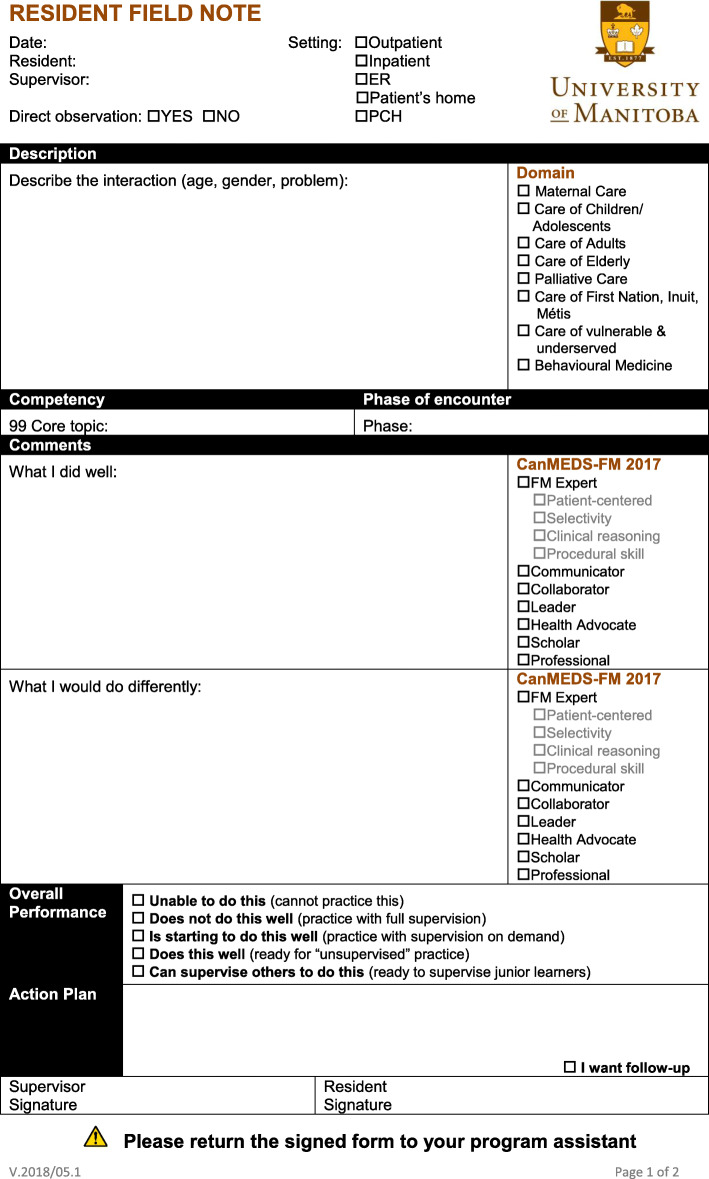
Field Note form for completion by residents in the Family Medicine residency program at the University of Manitoba

Although residents have always been provided the opportunity to review their progress with their preceptors, prior to implementing FNs, they were not required to formally self-evaluate. One of the requirements for life-long learning is the ability to evaluate one’s knowledge gaps in order to formulate learning plans [15]; therefore, physicians must become skilled in self-reflection. The CFPC has identified FNs as “the core assessment activity for programmatic assessment” [1] of learners. As such, FNs (Fig. 1) were introduced into the Department of Family Medicine residency program in the 2012–2013 academic year as a pilot project and were designed to incorporate the essential communication and management skills as outlined by the CFPC [1]. From an initial analysis of the data collected during initial FN implementation, Cavett, Halas, and Jamieson identified several challenges and varied uptake, warranting a longitudinal study of FN use [16]. Embedded within this larger study examining the use of FNs and their impact on resident education, we conducted a more in-depth analysis of a subset of FNs from residents who completed their training. While prior studies have described positive results in the use of FNs as a learning tool in Family Medicine training [6, 7], this research pays particular attention to contextual differences that may affect the use and utility of FNs as a learning tool. The key objectives of this research will be to determine:

1. the frequency and trends in the use of FNs by residents and/or their supervisors over the training period and between urban versus rural sites,
2. the content (e.g., types of problems encountered) captured by FNs during the training period,
3. the extent of CFPC defined “skill dimensions” addressed in the residents’ clinical experiences,
4. the nature of their learning experiences, self-assessments and action plans.

## Methods

### Study design and sample

This study is a retrospective mixed method analysis of secondary data collected by the Family Medicine Residency program at the University of Manitoba. Upon residents’ completion of training, the program gathers all educational documents generated in each resident’s file for collation by the University. The FNs collected for use in this study were copied from these folders prior to being transferred to the university files.

FNs were de-identified prior to the start of this study by administrative personnel from the Department of Family Medicine. All study procedures were conducted in accordance with relevant guidelines and regulations once a review was completed and approval received from the University of Manitoba Health Research Ethics Board # HS22239 (H2018:402). Informed consent was obtained from all participants except for graduates who had already completed the training program, in which case de-identified data was available from a department database for analysis. Guidance regarding access and use of this dataset was provided by the Privacy Officer of the University of Manitoba.

The FNs analyzed in this study were generated by a random sample of 16 residents from among the 70 residents (who will be included in the overarching study) who completed their training in June 2018, yielding 520 field notes for analysis. The University of Manitoba’s Family Medicine residency program includes residents from a variety of streams broadly categorized as the Urban, Rural and Northern Remote streams. While residents from all streams receive well-rounded educational experiences required to become confident generalists, how they learn this material and the settings in which they practice differ. Urban stream residents complete the majority of their training in Winnipeg, while Rural steam residents train in diverse communities ranging in size from 1000 to 40,000 people [13, 14]. This sample is comprised of FNs written by 8 Urban and 8 Rural residents. No FNs written by residents from the Northern Remote stream were selected.

### Methods and data analysis

The FNs were analyzed using basic descriptive statistics (frequencies and means) for various domains including FNs generated, patient populations served, CanMEDS-FM roles demonstrated, and the CFPC Priority Topics [11] discussed between residents and preceptors. The data was then compared across the Urban and Rural residency streams.

A qualitative content analysis [17] of the written FNs was conducted with a focus on the residents’ descriptions of their learning experiences as well as their self-assessments and action plans for future practice. Inductive content analysis was used for sections of the FN where residents responded to open-ended questions regarding what they did well in the clinical interaction, what they would do differently, and their proposed action plan (objective 4). FN content was imported onto NVivo 12 [18] and coded through First Cycle Coding employing Initial Coding, Evaluation Coding, and Process Coding principles, followed by Second Cycle Coding using Holistic Coding [17]. Content was then analyzed to explore and generate overall themes [19]. Coding was reviewed by two coders to ensure validity, while themes were reviewed and discussed for consensus by all three team members.

## Results

**Fig. 2:**
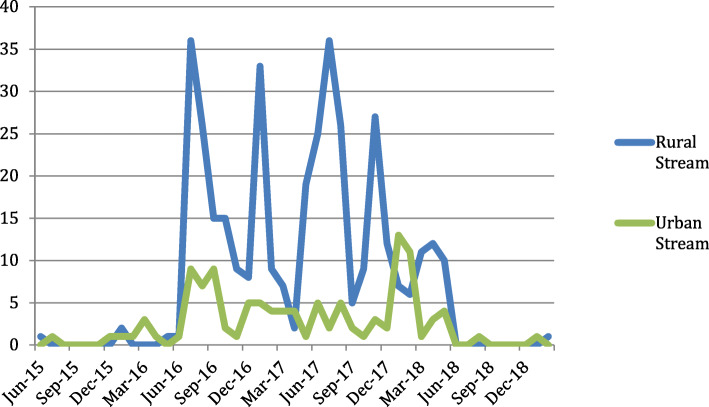
Number of field notes completed in Urban and Rural streams from June 2015 to February 2019. This includes 17 FNs from residents who were off time, that is began or completed the program outside of the official start and end dates, during their residency training (i.e. FNs completed before July 2016 and after June 2018). Note: This figure includes data from resident V1616. Removing the “super-user” (V1616) did not affect the cyclical variation in field note generation in the Rural stream

Analysis of the data demonstrated a wide variation in the total number of FNs generated by the residents during the course of their two-year residency program (range = 1–123). An average of 32.5 FNs was found for all 16 residents with a notable difference in the average numbers of FNs among residents in the Urban versus Rural streams (15 and 50 respectively). Of note, there was one Rural FN “super-user” (resident V1616) who generated roughly 31% of the FNs documented by all Rural residents; removing that resident from the data set decreased the Rural stream average to 39.6 FNs. There was also cyclical variability in the number of FNs generated over a two-year period in both Urban and Rural streams which aligned temporally, however peaks in FN generation were more pronounced in the Rural stream (Fig. 2). When the data from the aforementioned “super-user” resident was once again removed, the cyclical uptake among the Rural residents remained largely unchanged, however the Rural stream continued to have greater variability than among the Urban residents.

The top 10 most documented CFPC Priority Topics by the 16 residents are presented alongside the CFPC Top 10 (Table 1) [11]. Residents’ top 10 Topics address skin disorders, infections, and health maintenance whereas the CFPC Top 10 predominantly address chronic disease and mental health concerns (Table 1) [11]. Despite little agreement between the resident list and the CFPC Top 10, the Urban and Rural residents selected similar topics for review with 75% of their top 10 topics being identical (Table 2). Notably, no residents selected the following topics: Domestic Violence, Grief, Infertility, Lifestyle, Obesity, Parkinsonism, Rape/Sexual Assault and Somatization.

**Table 1:** Top 10 “99 Priority Topics” documented for all 16 residents compared to the CFPC Top 10 for residents to be competent [11]

Top 10 “99 Topics” Covered in Field Notes (***n*** = 16 residents)	CFPC Top 10 [11]
1) Skin Disorder	1) Depression
2) Infections	2) Anxiety
3) PHE/Screening and Well-Baby (Health Maintenance Visits)	3) Substance Abuse
4) Abdominal Pain	4) Ischemic Heart Disease
5) Depression	5) Diabetes
6) In Children	6) Hypertension
7) Pregnancy	7) Pregnancy
8) Joint Disorder	8) Headache
9) Chronic Disease	9) PHE/Screening
10) Low-Back Pain	10) Palliative Care

**Table 2:** Top 10 “99 Priority Topics” documented in the Urban and Rural streams

Urban (***n*** = 8 residents)	Rural (***n*** = 8 residents)
1) Skin Disorder	1) Skin Disorder
2) Abdominal Pain	2) Infections
3) Joint Disorder	3) In Children
4) PHE/Screening (Health Maintenance Visits)	4) Depression
5) Chronic Disease	5) Abdominal Pain
6) Difficult Patient	6) Pregnancy
7) Pregnancy	7) Well-Baby (Health Maintenance Visits)
8) Cancer, Depression, Infections, Low-Back Pain, Thyroid	8) Joint Disorder
	9) Anxiety, Dizziness, Elderly, Low-Back Pain

For both Urban and Rural residents (*n* = 8 for both streams), the most common documented Domains of Care were Care of Adults (Urban 56%; Rural 49%) followed by Care of the Elderly (Urban 22%; Rural 17%; Table 3). The least documented domains were Care of First Nations, Inuit, Métis; Care of Vulnerable & Underserved; and Behavioural Medicine (range 0–3.8%).

**Table 3:** Domains of Care encountered by Urban and Rural residents in FNs

Domain of Care	Urban (***n*** = 8 residents)	Rural (***n*** = 8 residents)
Maternal Care	4 (4.7%)	15 (4.4%)
Care of Children/Adolescents	11 (13%)	74 (22%)
Care of Adults	48 (56%)	166 (49%)
Care of Elderly	19 (22%)	57 (17%)
Palliative Care	1 (1.2%)	1 (0.3%)
Care of First Nations, Inuit, Métis	0 (0%)	1 (0.3%)
Care of Vulnerable & Underserved	2 (2.4%)	11 (3.3%)
Behavioural Medicine	0 (0%)	13 (3.8%)
**Totals**	**85**	**338**

The two CanMEDS-FM roles assessed most frequently for both streams were Communicator (Urban 29%; Rural 15%) and FM Expert- Clinical Reasoning (Urban 18%; Rural 20%; Table 4). The least reported roles overall for both streams were FM Expert – Procedural Skill (Urban 5.1%; Rural 3.5%), Leader/Manager (Urban 4.1%; Rural 3.3%), and Professional (Urban 3.6%; Rural 9.5%). Within both streams, the CanMEDS-FM roles recognized as being performed well were overall recorded much more frequently than those that could be improved.

**Table 4:** CanMEDS-FM roles documented as “Did Well” and “Would Do Differently” in FNs for Urban and Rural residents

CanMEDS-FM Roles	Urban (***n*** = 8 residents)			Rural (***n*** = 8 residents)		
	Did Well	Would Do Differently	Total	Did Well	Would Do Differently	Total Roles
FM Expert – Patient-Centered	20 (15%)	8 (13%)	28 (14%)	150 (17%)	8 (5.5%)	158 (15%)
FM Expert – Selectivity	5 (3.8%)	0 (0%)	5 (2.5%)	113 (13%)	18 (12%)	131 (13%)
FM Expert – Clinical Reasoning	23 (17%)	12 (19%)	35 (18%)	166 (19%)	36 (25%)	202 (20%)
FM Expert – Procedural Skill	5 (3.8%)	5 (7.8%)	10 (5.1%)	24 (2.7%)	12 (8.2%)	36 (3.5%)
Communicator	41 (31%)	16 (25%)	57 (29%)	137 (16%)	20 (14%)	157 (15%)
Collaborator	13 (9.8%)	2 (3.1%)	15 (7.6%)	58 (6.6%)	6 (4.1%)	64 (6.2%)
Leader/Manager	3 (2.2%)	5 (7.8%)	8 (4.1%)	23 (2.6%)	11 (7.5%)	34 (3.3%)
Health Advocate	11 (8.3%)	7 (11%)	18 (9.1%)	71 (8.1%)	7 (4.8%)	78 (7.6%)
Scholar	7 (5.3%)	7 (11%)	14 (7.1%)	43 (4.9%)	26 (18%)	69 (6.4%)
Professional	5 (3.8%)	2 (3.1%)	7 (3.6%)	96 (11%)	2 (1.4%)	98 (9.5%)
**Totals**	**133**	**64**	**197**	**881**	**146**	**1027**

In the sections of the FNs where responses to open-ended questions were recorded, 515 addressed “Did Well”, 363 addressed “Would Do Differently”, and 227 discussed “Action Plans.” Based upon these narrative comments, residents are able to form Action Plans, which are formulated through discussion between the resident and preceptor and identify the means by which a resident will address a particular learning gap. These may include reminders to review certain management guidelines or to implement a particular communication tool in patient and inter-provider encounters. Positive feedback (“Did Well”) primarily addressed the domains of history-taking (26%), physical exam skills (25%), and management (25%), whereas constructive comments (“Would Do Differently”) addressed management (26%) and physical exam skills (18%) as well as general knowledge and skills (for example, “ensure documentation clear. Review well child advice” [D2323]) (33%). The action plans were analyzed in terms of learning domains, where feedback was directed toward a need to increase knowledge (64%) and improve skills (32%), with very few recommending changes in attitude (3.4%). A thematic content analysis of these sections within the FNs can be described in terms of four emergent themes that describe residents’ performance: Patient-Centered Care, Patient Safety, Achieving Balance, and Confidence Within each of these themes, comments reinforcing positive aspects of residents’ performances and remarks on areas of improvement were identified (Table 5).

**Table 5:** Further examples from FNs illustrating the four themes

Theme	“Did Well” Examples	“Would Do Differently” Examples
Patient-Centered Care	“approached patient in nonjudgemental (sic) manner, built rapport, promoted transparency and honesty” (T5540) “explored deeper issue for appt [appointment]... Motivational interviewing - “what can we do to help you”. “What kind of help do you need now.”” (R5630) “Patient led management plan” (A1278)	“…suggest exploring illness experience more…” (B1009) “develop confidence in having conversations surrounding narcotics and adhere to strict prescribing principles…” (X5650) “Break explanation into ruling out “scary stuff” then getting into less common causes” (B1009) “Be careful not to become to “paternal” in approach. Always listen to concerns” (S0910)
Patient Safety	“Evaluated old chest and current presentation to determine ddx [differential diagnosis] and treatment plan” (A1278) “realized this is more sinister than a ‘drug rash’ and requires more extensive work-up.” (R5630) “distinguished that the newborn was not in critical condition or needing urgent care” (A1278)	“maintain a good differential and high index of suspicions for non healing wounds (ex. Suspect fistula early)” (G1010) “careful not to rely on the evaluation of our patients by other health care professionals. Always do my own evaluation to validate concerns. In this way, diagnoses will be checked thoroughly and not missed” (F8888)
Achieving Balance	“Is comfortable being independent, but quick to know limits.” (R5630) “calm confident manner put the patient at ease.” (Z1290)	“be less scared to call specialist/city If no answer go through emerg rather than wait” (G1010) “Sense of insecurity apparent to patient on the phone - I was trying to present info in a nonbiased way but came across as just being unsure at what we should do” (T5540)
Confidence	“recognized anaphylaxis as an emergency. Initiated treatment” (A1278) “Ruled out red flags allowing safe d/c [discharge] w/out exact dx [diagnosis]” (Z1290)	“trust my clinical judgement (sic) on sickness of patient.” (A1278) “gain more comfort in assessment of fetal lie and use of Doppler for FHR” (X5650) “gain further confidence in definitive care plan. IE: scope or not to scope.” (Z1290)

The theme of *Patient-Centered Care* refers to the approach residents used to foster open communication, trust, and understanding which enabled them to fully explore patients’ perspectives while respectfully and collaboratively providing care in patients’ best interests (for example, “explored deeper issue for [appointment]...” [R5630]). In contrast, feedback for improvement addressed residents’ skill and confidence in conducting difficult conversations and exploring patients’ illness experiences in greater depth (for example, “Break explanation into ruling out “scary stuff” then getting into less common causes” [B1009]).

The theme of *Patient Safety* describes how residents recognized issues where patient safety was at risk and how the need to ensure patient safety impacted residents’ assessments, clinical reasoning, and decision-making (for example, “realized this is more sinister than a ‘drug rash’ and requires more extensive work-up.” [R5630]). In contrast, comments for improvement identified situations where residents needed to think more broadly in order to ensure nothing dangerous was overlooked (for example, “maintain a good differential and high index of suspicions for non-healing wounds (ex. Suspect fistula early)” [G1010]).

The theme *Achieving Balance* conveys situations where residents functioned independently while also recognizing their own limits through asking for assistance when needed, as well as times when they balanced both remaining professional while demonstrating empathy (for example, “calm confident manner put the patient at ease.” [Z1290]). Comments for improvement demonstrated how residents needed to strike a balance between being cautious and trusting their knowledge in order to collaborate with patients and other health care providers (for example, “be less scared to call specialist/city. If no answer, go through emerg rather than wait” [G1010]).

Finally, the theme *Confidence* focuses on residents’ attitudes in their assessments, clinical reasoning, and decision-making and how they affected the next steps they took in patient care. In writing, “ruled out red flags allowing safe [discharge] w/out exact [diagnosis]” (Z1290) this participant demonstrated confidence in his assessment and reasoning. Similarly, “trust my clinical judgment (sic) on sickness of patient.” (A1278) demonstrated the need to be more confident in the accuracy of his/her assessment.

## Discussion

### Field note use and user perceptions

This study examined the content of FNs generated by a cohort of residents from Urban and Rural Family Medicine streams to gain a better understanding of the content of FNs and how residents reflected on their progress and future learning needs. It is evident from the findings that there is great variability in FN use between streams. In addition to considering the structural factors and dynamics that may influence FN use, we offer a theoretically informed interpretation of the results.

Within this sample of residents, FN generation was three times greater in the Rural stream compared to their Urban counterparts. This may be due to the structural differences between the two training programs. The Urban stream and the majority of the Rural streams have set Family Medicine Block Time (FMBT) completed in Family Medicine teaching units with additional specialty-based rotations interspersed between the blocks [13, 14]. However, one of the Rural stream sites provides an integrated program in which FMBT is integrated with other clinical domains throughout residents’ training (i.e. no separate FMBT) [13] and as such, residents in this stream have more interactions with family medicine preceptors, theoretically allowing for more opportunities to generate FNs. These structural differences, in addition to the variations in FN generation between streams, present a topic for future investigation with respect to how the structure of Family Medicine programs supports and encourages reflective feedback. Further qualitative investigation of structural and motivational factors among residents and their supervisors would enhance our understanding of the varying uptake and marked decreases of FN generation over the course of the program (Fig. 2). This type of inquiry would also be beneficial in further understanding circumstances influencing a “super user” resident.

The overall uptake and content of FNs may be influenced by how users perceive the overarching purpose of FNs. Some residents may perceive FNs as a self-reflection tool for their performance for that specific day rather than a means to facilitate ongoing self-reflection. Furthermore, some residents may simply interpret FNs as a personal tool for single use, rather than as an instrument for informing comprehensive assessment. The same may be said for educators who may view FNs as a tool to foster discussion about a particular patient encounter rather than using the notes to chart residents’ progress over the course of their program. This “one-time” perspective risks impeding residents’ ongoing self-reflection and preceptors’ longitudinal assessment of residents’ development [5]; similarly areas identified as needing improvement may be overlooked if not re-visited in future evaluations [5]. In addition to using FNs on a day-to-day basis to foster discussion and formative feedback, regularly reviewing series of FNs may identify underlying areas for improvement. This may provide the program, preceptors, and learners with insight into areas where more longitudinal focus is required.

FNs enable the assessment of easily observed skills such as communication, procedural skills, and medical knowledge, while more latent, deep-seated skills such as professionalism and leadership may be more challenging to evaluate and as such may be overlooked. While FNs contain lists of CanMEDS-FM roles that can be marked as “done well” or “requires improvement”, it is essential that this assessment is supported by narrative feedback from both learners and preceptors [1, 5]. In this way, specific positive aspects of a resident’s performance will be reinforced, while skills that require improvement can be explored through particular examples. For example, while a resident could tick off the checkbox next to “Collaborator” as a skill they wish to improve (Fig. 1), it would be much more valuable to be provided with specific feedback on how well they communicated during an encounter. An example in this study of the provision of targeted feedback includes constructive commentary provided by a preceptor to one resident encouraging them to be less intimidated to call a specialist (Table 5). In this way, less overt skills can be discussed and evaluated through individual and relevant experiences which will have a positive impact on a resident’s performance in the future.

Interviews with both residents and educators may aid in clarifying current perspectives on FN use and thus address these possible inconsistencies. Overall, the objective of FNs should be clearly expressed to both residents and educators in the future in order to maximize their efficacy as either learning or evaluative tools (or both) within the Department of Family Medicine. Additionally, residents should be encouraged to provide detailed self-reflection on their performance without fear of penalization. This will allow for optimization of the use of FNs as a tool to guide residents through areas of unfamiliarity and discomfort onto higher levels of learning.

### Self-perception in the learning environment

Within the FN documentation in this study, some CFPC Topics, CanMEDS-FM roles, and Domains of Care were recorded more frequently, while others were documented less frequently. The phenomenon of variable documentation of CanMEDS-FM roles is not unique to this study. Mathew, et al., found that the roles of Clinical Expert and Communicator were assessed most frequently, with Professional being assessed less commonly and Leader not being assessed at all [7]. Considering what is recorded less frequently, the complexity and psychosocial nature inherent in these areas was noted. By neglecting to address complex competencies, residents may have missed opportunities for growth.

The presented results indicating variable use and the tendency to address more simple issues suggest potential convergence in the concepts of comfort and building competence as possible influential factors shaping the content of FNs and residents’ motivations to share their challenges and learning goals. The idea that learners’ comfort and competence may influence educational advancement was presented by Vygotsky’ in his theory of learning. Vygotsky identified three learning zones: a central “zone of comfort” where learners feel comfortable in their knowledge and skills, the Zone of Proximal Development (ZPD) surrounding this where learners move into the unknown with support and mentoring to grow in confidence and competence, and the “zone of danger” (outside the ZPD) where fear and loss of control inhibit learning [20]. By choosing to focus on domains where residents already feel competent, residents may remain in the central zone and miss out on the opportunity for further progression. Only by challenging themselves with clinical topics with which they are uncomfortable, will residents grow in confidence and competence.

Vygotsky’s theory may also be used as a framework to explore residents’ motivations for reflection upon certain topics and to hypothesize how residents’ self-image and comfort may impact their educational advancement. During Family Medicine residency, residents must master a vast amount of knowledge and skills in a short period of time. They must not only acquire this knowledge, but also confidently apply it in everyday practice when making medical decisions. Despite being at an early stage in their professional career, residents are expected by patients and educators alike to demonstrate relatively high degrees of certainty as practitioners while simultaneously navigating their roles as learners. As such, there may be an internal struggle for residents as they balance the roles of both physician and learner [21–23]. This in turn may influence residents’ self-confidence and self-perception and impair their progress from areas of certainty through the ZPD to areas of unfamiliarity where learning might be more enriched [23].

Throughout this study, this phenomenon appears to be evident in that certain competencies, particularly those addressing complex psychosocial issues, were selected less frequently than other competencies. For example, of the 99 CFPC Priority Topics, none of the FNs documented the following topics: Domestic Violence, Grief, Infertility, Lifestyle, Obesity, Parkinsonism, Rape/Sexual Assault and Somatization. Additionally, the CanMEDS-FM roles of Communicator and Clinical Expert have been assessed more commonly than the Professional and Leader roles. Another example of how this theory has manifested is that in the narrative portions of FNs, residents reflected upon their communication and clinical reasoning skills*,* areas they may be more familiar assessing, more often than they discussed their ability to strike a balance between practicing independently and recognizing limits in their own capabilities. This may indicate a preference in reflecting upon more overt skills that were likely previously assessed by supervisors rather than assessing their leadership skills, a role they may not completely identify with at this point in their professional development.

These early findings may indicate increased comfort in reflecting upon areas of familiarity while avoiding topics that are out of residents’ comfort zones. As such, they may feel reluctant to reflect upon new experiences considering these are areas where they may feel less confident. With the breadth of knowledge and skills generalists are required to obtain, residents may doubt their abilities at such an early stage in their careers [23]. If learners’ decreased comfort and confidence are in fact contributing factors for avoiding reflection upon certain competencies, it may also be seen as a barrier to residents’ movement through the ZPD onto more unfamiliar areas of learning [20]. Further exploration of these questions, ideally through interviews with learners, is needed.

### Implications

Avoidance of unfamiliar or difficult topics and domains may impede honest self-reflection and residents’ overall development into well-rounded and confident attending physicians. Educators must aim to foster a safe and encouraging learning environment that optimizes education while providing safe patient care. Only within a supportive training environment will learners feel they can honestly evaluate and debrief their performance with preceptors without fear of judgement or negative evaluation. It is imperative to encourage collaboration between residents and preceptors in pushing the boundaries of residents’ comfort zones in order to learn and reflect on the necessary skills, both in knowledge and application, required to become competent and confident generalists.

### Limitations

This project analyzed a subset of FN data from an overarching study. In the overall project, the resident sample will be from the Urban, Rural, and Northern Remote streams whereas this inquiry only analyzed data from Urban and Rural residents. This limits the generalizability of the findings. Secondly, one participant within the Rural stream generated a significant number of FNs, which may have skewed the data from that stream, while simultaneously highlighting differences in FN uptake. Thirdly, residents typically only generate one FN per day regardless of the number of patient encounters, thus the data may not reflect the variety of patient care experienced in day-to-day practice reflecting only what residents choose to document. Finally, factors influencing residents’ selection of FN topics were not captured by the study design.

## Conclusion

Differences in FN generation were found between the Urban and Rural streams, while both streams demonstrated cyclical variation over the course of the two-year residency. More in-depth analysis of FN content revealed particular medical encounters and CanMEDS-FM competencies were more commonly reflected upon, while residents did not frequently create FNs which addressed challenging psychosocial topics or marginalized or vulnerable populations. Vygotsky’s work on learners’ attainment of competence is proposed to explain why learners did not explore challenging or unfamiliar topics. By remaining close to the zone of comfort, learners miss valuable learning opportunities, impeding their professional development.

The responsibility of providing patient care during residency training is difficult to balance with one’s self-assessed knowledge deficits or weaknesses, especially considering the breadth of knowledge and skills required for generalist care. It is essential that feedback regarding the perceived use and effectiveness of FNs in the Family Medicine residency program is obtained and considers discomfort or unfamiliarity with certain topics, within a safe environment, to foster growing confidence and competence.

Further research is needed to explore why some residents and preceptors become “super-users” of FNs compared to others, and how the learning environment may impact the completion of FNs by residents. As such, both quantitative and qualitative research, along with discussions with both learners and educators alike, should be undertaken to fill in these knowledge gaps in order to better support Family Medicine residents throughout their training. Overall, it is essential for programs to be cognizant of how the learning environment can influence residents’ engagement in self-reflection. It is vital that programs and residents collaboratively take steps to address any obstacles to honest self-assessment and acquisition of constructive feedback in order to ensure the competence of residents by the end of the program.

## Data Availability

Data at the level of individual learners is not available as participants only provided consent for aggregate findings to be disseminated. For requests to review the data from this study, please contact Dr. Teresa Cavett (teresa.cavett@umanitoba.ca).

## Abbreviations

FN(s): Field Note(s)
CBME: Competency-Based Medical Education
CFPC: College of Family Physicians of Canada
ZPD: Zone of Proximal Development
